# Undermining racism: A road to promoting equity in oral health

**DOI:** 10.1111/jphd.12511

**Published:** 2022-06-21

**Authors:** Michael Monopoli, Ifetayo B. Johnson

**Affiliations:** ^1^ CareQuest Institute for Oral Health Boston Massachusetts USA; ^2^ Oral Health Progress and Equity Network (OPEN) Washington District of Columbia USA

**Keywords:** capacity building, CareQuest Institute, health equity, OPEN, oral health, social determinants of health, social networks, systemic racism

## Abstract

**Background**: The inequities caused by racism include negative impacts on health and quality of life. A key grant‐making strategy of the CareQuest Institute for Oral Health has been the development of a network of stakeholders, the Oral Health Progress and Equity Network (OPEN), which engages and leverages community voices to address racism and the corresponding health inequities across the lifespan.

**Methods**: OPEN's nearly 3000 members undertake various approaches to acknowledge and address the negative impact of systemic racism on health. OPEN has developed structures and offerings that have advanced a unique culture of equity, which encourages authentic dialogue. OPEN created and facilitated cohorts within the network called Network Response Teams to elevate awareness and develop strategies to address health disparities advocating for specific populations, including LGBTQIA+, Native American, LatinX, People with Disabilities, and rural residents, Medicare, and Medicaid. Network members work toward an equitable oral health system by aligning common goals.

**Results**: The activities and products of these teams and OPEN developed trusted relationships to combat the impacts of racism and promoted equitable oral health locally and nationally.

**Conclusion**: Supporting network development is a highly effective tool to combat racism.

## INTRODUCTION

A wicked problem, according to Horst Rittel [1], is a social or cultural issue that is difficult to explain and inherently impossible to solve. Examples include hunger, income disparities, and systemic racism. To address wicked public health problems, some grant‐makers support collective action by multiple stakeholders who are members of a particular *network*, a group of interconnected people and organizations working together to achieve common goals.

The CareQuest Institute for Oral Health (https://www.carequest.org), a nonprofit organization committed to building a future in which everyone can reach their full potential through optimal health, is a pioneer in this approach to grant‐making. Since 2011, the CareQuest Institute for Oral Health and its predecessor, the DentaQuest Foundation, have supported the development of a systems change network, the Oral Health Progress and Equity Network (OPEN), to address the structural determinants of persistent oral health inequities in the United States. [2].

Grant‐makers can play several important roles in the development and support of such networks. These typically include the following:

- Catalyst: establishes purpose or vision.
- Sponsor: provides resources for knitting, organizing, growing.
- Weaver: increases connections among participants and grows the network by connecting to new participants.
- Coach: provides advice, as needed.
- Participant: participates without a direct leadership role.
- Assessor: diagnoses network needs and recommends next steps [3].

The CareQuest Institute for Oral Health has, since 2011, played each of these roles in support of OPEN, whose membership has grown to almost 3000 members working in oral health advocacy, care, education, public health, public policy, community‐based services, and related fields. There is no charge to join the network, and all its offerings are free to members.

Since its launch, OPEN has explored and addressed the role played by structural disparities and systemic racism in perpetuating inequitable access to oral health. Recognizing that a healthy mouth is critical to overall health and the ability to thrive, OPEN has evolved from a group of national organization leaders working to improve oral health care delivery to a national network of individuals working at the community, state, and national levels to improve timely access to appropriate, quality care, largely by addressing the root causes of disparities, including systemic racism. The remainder of this article describes the growth, work, and success of this network in addressing systemic racism.

## THE PROBLEM

Oral health disparities in America are well‐documented [4, 5]. However, the isolation of oral health from overall health in research, coverage, and care diminishes the gravity of oral health disparities in national conversations about health care and systemic racism. To improve oral health outcomes, systemic racism must be confronted. The Centers for Disease Control (CDC) states that the pervasiveness of systemic racism has a “profound and negative impact on communities of color … placing those within these populations at greater risk for poor health outcomes.” [6, 7]. The negative impact of systemic racism, however, affects us all and degrades the quality of life of all Americans.

As described above, wicked problems are intractable interconnected problems that are difficult to define or solve. The more complex the problem is, the more challenging it is to address. Few problems are more wicked than racism and its devastating impact on health [8]. The insidious nature of racism may compel those least impacted by it to ignore its existence and to circumvent their discomfort by retreating into “safer” topics.

Consequently, our health systems tend to favor the status quo without acknowledging that it was not designed to include those who first occupied this land, or have a different orientation, or have more melanin. Systems designed for the comfort of a particular group negate the needs of others. This is not a question of “good” or “bad” people but rather of good and bad systems.

Bad systems fail to recognize, for example, that “essential workers” who are paid the least, have the least amount of flexibility in their workday, and may have to take three buses to get to a dentist, may accordingly be far more likely to forego a dental cleaning or appointment to address dental pain. They are also less likely to have dental coverage or the ability to pay for extensive dental work out of their pocket. However, few dental offices offer flexible hours or payment methods, and fewer dentists can afford to accept Medicaid [9]. It is a system described by Dr. Caswell Evans, Associate Dean for Prevention and Public Health Sciences, University of Chicago, as one in which those most in need are least likely to be served, and those least in need are most likely to be served. These failed systems have resulted in devastating disparities.

For example, untreated decay in Pine Ridge Reservation Native American adults has been as high as 97 percent; nearly twice as many non‐Hispanic Black or Mexican American adults have untreated cavities as non‐Hispanic, white adults; low‐income or uninsured adults are three times more likely to have four or more untreated cavities than adults with higher incomes [10]. These disparities are created by inequitable systems, and systems can be changed for the benefit of all.

In *The Sum of Us: What Racism Costs Everyone and How We Can Prosper Together*, Heather McGhee argues that racism has a devastating influence on everyone, and our society grows and prospers in health and wealth by confronting and working to eliminate racism. She states that we are all lacking the best things, “This includes the white Americans who are the largest group of the uninsured and the impoverished as well as the Americans of color who are disproportionately so” [11].

McGhee recounts an incident where a southern town built a grand, resort‐style pool in the 1950s, and post desegregation, they chose to fill it with dirt and close the parks rather than integrate. This meant, of course, that poor and middle‐class white people did not have a pool or park either. Racism that affects poor people of color impacts poor white people in larger total numbers [12]; thus, eradicating systemic racism in health care and society is in the best interest of all Americans.

The very word “racism” elicits an immediate visceral response; generally, a desire to turn away to avoid the discomfort of the ensuing conversation. However, attaching a modifier as OPEN does makes conversations less awkward. OPEN sessions discuss and examine *systemic* or *institutional* racism, rather than *individual* racism. This shifts blame from the person to the systems of inequity to which everyone, to varying degrees, is subjected. It creates a “safe” space for individuals to share their stories and fosters understanding and compassion. Creating a nurturing environment in which racism can be confronted safely, openly, and regularly is the work of OPEN.

## OPEN'S APPROACH TO TACKLING RACISM

In 2011, a small think tank of national oral health leaders convened to improve access to oral health and concluded that equitable oral health was a social justice issue. The think tank consisted of approximately 20 members, including representatives from the American Dental Association, National Dental Association, Center for Disease Control, Health and Human Services, American Academy of Pediatrics, Henry Schein, Pew Charitable Trust, universities, dental plans, and state associations [13]. With funding from CareQuest, the think tank expanded to support 20 initiatives in areas with high oral health disparities and to advance the sharing of best practices and collaborative learning through a variety of national and regional meetings throughout the year. OPEN emerged from this nationwide conversation, which now engages policymakers, providers, public health activists, and grassroots organizers working together for an equitable healthcare system.

In 2015, OPEN reimagined ways to become a stronger equity champion. Both CareQuest and the network's members recognized the need to elevate the voices of community‐based and grassroots organizations in OPEN. In 2016, CareQuest allocated funding to engage grassroots and state representatives from all 50 states in OPEN convenings. These new voices were instrumental in strategic planning, providing insights into the local impacts of health inequities and their underlying causes, and led to a vibrant exchange of ideas and best practices.

This inclusiveness provided a rare opportunity for bidirectional communication between local and national stakeholders. Occasionally, this resulted in challenges to the status quo and confrontations regarding the network's commitment to equity. For example, during debates over the inclusion of dental benefits in publicly funded health coverage, compassionate grassroots leaders would sometimes challenge traditional practitioners' responses to patients in need. At other times, altruistic community leaders had to face the reality of clinicians' astonishing costs of running a dental office and paying school debt.

Honest and uncomfortable conversations about health disparities, racism in healthcare, and social justice moved the debate from blaming individuals to examining a system that neither adequately compensated providers nor provided comprehensive coverage to the patient. This awareness of the role of systemic racism in driving disparities brought the network into greater alignment and increased collective efforts to address systemic racism. The network collectively identified 5‐year goals for measurable system change that its members pursued collaboratively [14].

As more advocates joined OPEN, the network's calls to confront racism increased. In response, every major OPEN meeting included at least one, and often more, sessions on equity and racism. Conference participants received pre‐readings and videos on equity, social justice, and/or racism, which were explored in small‐group discussions at meetings [15, 16, 17].

OPEN members from various backgrounds learned to talk candidly with one another. Black, White, Christian, Jewish, Muslim, Latin, Native American, Asian, East Indian, LGBTQIA, rural, urban, and disabled members each brought their own perspectives. This caused awkwardness, which members typically agreed to work through rather than avoid. Trained staff guided authentic and fruitful conversations. OPEN also actively engaged in building relationships, which was necessary for fostering risk‐taking in conversations. OPEN members expected all convenings to have an equity‐centered section where the historical context of systemic racism was shared, followed by small‐group discussions. OPEN confronted the wicked problem at every convening, and the members' comfort levels slowly increased.

Many network members expressed an interest in exploring systemic racism further, but others were hesitant. The network and staff encouraged personal discussions, webinars, readings, and book clubs. These activities helped reluctant members feel more comfortable in the antiracism space. Likewise, network leadership and staff members committed to fostering deeper conversations about racism, and using an equity lens during their meetings, laying a strong foundation for forward momentum.

Racism is depressing, but as Dr. Edith Eger wrote in her book, *The Gift*, “the opposite of depression is *expression*. What comes out of you doesn't make you sick, what stays in there does” [18]. Cohesion grew as the network summoned the courage to face race, discuss racism, and collaboratively address the root causes plaguing our country.

To further build equity capacity and strengthen connections among members, OPEN created Network Response Teams (NRT). OPEN NRTs are action/learning communities that address equity and specific topics of interest for marginalized groups. OPEN NRTs focus on populations such as Native American, LGBTQ+, Latinx, Rural, and People with Disabilities, as well as oral health‐related topics such as Medicare, Medicaid, public perception, policy, and data.

Honing their focus on equity, representatives from each NRT came together in 2020 to form a Health Equity “Super NRT.” The purpose of the Health Equity NRT is to ensure that all work done by OPEN:

- is conducted through an equity lens;
- uses the Jemez Principles for Democratic Organizing [19], a set of six principles to help ensure equity, by committing to which participants agree to be inclusive, emphasize bottom‐up organizing, let people speak for themselves, work together in solidarity and mutuality, build just relationships among themselves, and commit to self‐transformation; and
- adheres to OPEN's Justice, Equity, Diversity, and Inclusion (JEDI) statement [20].

OPEN members design network meetings using OPEN's equity checklist, and participants are surveyed afterward to ensure that they feel welcomed, heard, and supported. The Health Equity Super NRT also conducted an equity assessment of the network to ensure that OPEN leadership and members adhered to these guidelines. We must “practice what we preach”! Oral health serves as a central focus for the network, but it is the alignment between equity and social justice that ignites and binds the network's work.

It took several years, a pandemic, and witnessing the murder of George Floyd to nudge network members to expand the half‐day‐long equity conversations to more in‐depth discussions on the impact of racism and health disparities [21]. OPEN created toolkits and elicited the expertise of numerous speakers in the field of racial healing, including Dr. Gail Christopher, Dr. Camara Jones, Debby Irving, Jonathan Metzel, and Heather McGhee. Their presentations provided the language, historical background, and systemic perspectives needed to delve into tough racial topics, including redlining, implicit bias, white supremacy, health disparities, social determinants of health, and institutional racism. Network members subsequently began serious explorations of the historical events and systems that created our present‐day inequities. This work yielded a brave and safe space where participants could hold otherwise dangerous conversations.

In 2021, OPEN mapped the national oral health system and identified five leverage points to create equitable systems change: amplifying consumer voices, advancing oral health policy, dental/medical integration, emphasizing prevention over intervention, and bringing care to people. In 2022, network members will identify and flesh out strategies to advance these levers. The work continues.

## ENVISIONING THE FUTURE

Racism is deeply rooted in America, going as far back as its founding years. In 1619, the *White Lion* sailed to the shores of Jamestown, Virginia, with 20 enslaved Africans stolen from a Portuguese slave ship [22]. Our country's founding documents strove to establish a more perfect union, one in which justice, domestic tranquility, and general welfare would secure the blessings of liberty. However, numerous constitutional amendments were required to address issues of inherent racism and inequity, including the abolition of slavery, equal representation and protection under the law, the right of all men to vote regardless of color, and the right of all people to vote regardless of gender [23]. The struggle against systemic racism is an unwelcome reminder that our health system is riddled with inequities that shall persist until we collectively alter beliefs and behaviors among professionals, patients, politicians, healthcare systems, and the public. It also provides hope that a more perfect union may be possible.

OPEN's response to racism is to treat the word “network” as a verb rather than a noun. We work to recognize systemic racism, address it head on, build relationships around honest dialogue, carefully frame our messages, confront the discomfort that may ensue, and never give in to despair.

OPEN welcomes you to join its efforts to eradicate racism and achieve equitable oral health for all. Join by registering on the OPEN online platform, www.communities.openoralhealth.org.
